# Supplementary material for: A tale of osteoarthritis among older adults during the COVID-19 pandemic in Bangladesh: A repeated cross-sectional study
Source: PLoS One. 2022 Sep 20;17(9):e0274838. doi: 10.1371/journal.pone.0274838 (PMC9488814; doi:10.1371/journal.pone.0274838)
Supplement: Attachment — Submitted filename: Review response_R1.docx [file pone.0274838.s002.docx]

Response to Reviewers’ Comments - A tale of osteoarthritis among older adults during the COVID-19 pandemic in Bangladesh: A repeated cross-sectional study

Thank you for giving us the opportunity to revise and resubmit the manuscript and for the constructive comments. In the following table, we include the reviewers’ comments and outline our changes in responses to each comment. We have systematically incorporated reviewers’ comments and suggestions, which has enhanced the manuscript. We are confident that the revisions have improved the quality of our manuscript.

**Journal requirements**

| **Comment/Suggestion** | **Authors’ Responses** |
| --- | --- |
| Please ensure that your manuscript meets PLOS ONE's style requirements, including those for file naming. The PLOS ONE style templates can be found at https://journals.plos.org/plosone/s/  file?id=wjVg/PLOSOne_formatting_sample_main_body.pdf  and  https://journals.plos.org/plosone/s/  file?id=ba62/PLOSOne_formatting_sample_title_authors_affiliations.pdf | We ensure that the manuscript meets PLOS ONE's style requirements. |
| 2. Thank you for stating the following financial disclosure:  "No"  At this time, please address the following queries:  a) Please clarify the sources of funding (financial or material support) for your study. List the grants or organizations that supported your study, including funding received from your institution.  b) State what role the funders took in the study. If the funders had no role in your study, please state: “The funders had no role in study design, data collection and analysis, decision to publish, or preparation of the manuscript.”  c) If any authors received a salary from any of your funders, please state which authors and which funders.  d) If you did not receive any funding for this study, please state: “The authors received no specific funding for this work.”  Please include your amended statements within your cover letter; we will change the online submission form on your behalf. | We have added the statement ‘The authors received no specific funding for this work’. We have also added this in the cover letter. |
| 3. Thank you for stating the following in your Competing Interests section:  "No"  Please complete your Competing Interests on the online submission form to state any Competing Interests. If you have no competing interests, please state "The authors have declared that no competing interests exist.", as detailed online in our guide for authors at http://journals.plos.org/plosone/s/submit-now  This information should be included in your cover letter; we will change the online submission form on your behalf." | We have added the statement, “The authors have declared that no competing interests exist”. We have also added this in the cover letter. |
| 4. In your Data Availability statement, you have not specified where the minimal data set underlying the results described in your manuscript can be found. PLOS defines a study's minimal data set as the underlying data used to reach the conclusions drawn in the manuscript and any additional data required to replicate the reported study findings in their entirety. All PLOS journals require that the minimal data set be made fully available. For more information about our data policy, please see http://journals.plos.org/plosone/s/data-availability.  "Upon re-submitting your revised manuscript, please upload your study’s minimal underlying data set as either Supporting Information files or to a stable, public repository and include the relevant URLs, DOIs, or accession numbers within your revised cover letter. For a list of acceptable repositories, please see http://journals.plos.org/plosone/s/data-availability#loc-recommended-repositories. Any potentially identifying patient information must be fully anonymized.  Important: If there are ethical or legal restrictions to sharing your data publicly, please explain these restrictions in detail. Please see our guidelines for more information on what we consider unacceptable restrictions to publicly sharing data: http://journals.plos.org/plosone/s/data-availability#loc-unacceptable-data-access-restrictions. Note that it is not acceptable for the authors to be the sole named individuals responsible for ensuring data access.  We will update your Data Availability statement to reflect the information you provide in your cover letter.  Please review your reference list to ensure that it is complete and correct. If you have cited papers that have been retracted, please include the rationale for doing so in the manuscript text, or remove these references and replace them with relevant current references. Any changes to the reference list should be mentioned in the rebuttal letter that accompanies your revised manuscript. If you need to cite a retracted article, indicate the article’s retracted status in the References list and also include a citation and full reference for the retraction notice. | We have added the following line in the data availbality statement ‘All relevant data are within the manuscript and its Supporting Information files’,  and also added data as supplementary material.  We have also checked the reference list and corrected if necessary. |

**Reviewer 1**

| **Comment/Suggestion** | **Authors’ Responses** |
| --- | --- |
| Thanks to the authors for addressing an important issue and I am happy reading the paper. It is a well written manuscript and easy to understand the rationality.  The rationality is clear and well organized manuscript. I am happy reading the paper. | Thanks for appreciating our paper. |
| I don’t have much comments just quarries is the outcome variable we self reported?  What is the justification using self reported disease for a medical research? | Thanks very much for noting this important issue. Due to the restriction imposed due to COIVD-19 pandemic we could not collect data face to face and objectively measure osteoarthritis through radiographic evidence. We have acknowledged this as a limitation. Please also note that several medical research has been conducted remotely during the COVID-19 pandemic:  ^1^Chew, N. W., Wong, R. C., Djohan, A. H., Ngiam, J. N., Chai, P., Yeo, T. C., ... & Sia, C. H. (2021). The association of face mask use with self-reported cardiovascular symptoms during the COVID-19 pandemic. Singapore Medical Journal.  ^2^Imeri, H., Holmes, E., Desselle, S., Rosenthal, M., & Barnard, M. (2021). The impact of the COVID-19 pandemic on self-reported management of chronic conditions. Journal of Patient Experience, 8, 23743735211007693.  ^3^Ding, Q., Massey, D., Huang, C., Grady, C. B., Lu, Y., Cohen, A., ... & Krumholz, H. M. (2021). Tracking Self-reported Symptoms and Medical Conditions on Social Media During the COVID-19 Pandemic: Infodemiological Study. JMIR public health and surveillance, 7(9), e29413. |
| Please re edit the conclusion of the abstract it overtly written please be specific in writing the conclusion. | We have edited the conclusion of the abstract. Please see page 4 line 51 56 in the revised manuscript. |

**Reviewer 2**

| **Comment/Suggestion** | **Authors’ Responses** |
| --- | --- |
| The manuscript " A tale of osteoarthritis among older adults during the COVID-19 pandemic in Bangladesh: A repeated cross-sectional study is interesting. However, after reading the manuscript, I have certain reservations. | Thanks very much for reviewing our paper. |
| 1. Authors are advised to correct their grammatical mistakes throughout the whole manuscript. | We have checked for the grammatical mistakes throughout the manuscript and corrected where necessary. |
| 2. Incomplete sentences Under introduction lines 107-108. | We have revised the Introduction section to make it more clearer and coherent. |
| 3. Research gaps and objectives should be clearer. | We have revised the Introduction section to make the rationale of the research and objectives clearer. |
| 4. The inclusion criteria under the section ‘Study design and participants’ adopted age should be >= 60 to match the analysis if I am not wrong. | Corrected as you have suggested. Please see page 9 line 155. |
| 5. In table 1, the format of the >= symbol should be the same for all indicators. For age, you used >= 70 but for family size you used ≤4. | We have corrected this in all the tables |
| 6. Table 2 and the data presentation is not in the proper manner. Authors are advised to correct the table's format. | We corrected that format of Table 2. |
| 7. Authors are advised to show the results of binary logistic regression for all covariates, both for cOR and aOR, by creating a separate table. | We have now provided results of binary logistic regression in a separate table (Table 4) and narratives (please see page 18 line 258-267) |
